# A pH-sensitive, renewable invisible orthodontic aligners coating manipulates antibacterial and *in situ* remineralization functions to combat enamel demineralization

**DOI:** 10.3389/fbioe.2024.1418493

**Published:** 2024-07-22

**Authors:** Qi Qin, Wenhong Yuan, Jiarui Zhang, Yang Gao, Yanling Yu

**Affiliations:** ^1^ School of Stomatology of Qingdao University, Qingdao, China; ^2^ Qingdao Municipal Hospital, Qingdao, China; ^3^ Qingdao Jiaozhou Central Hospital, Qingdao, China; ^4^ Qingdao Stomatological Hospital Affiliated to Qingdao University, Qingdao, China

**Keywords:** white spot lesions, zwitterion, antimicrobial properties, remineralization, coating

## Abstract

During invisalign treatment, as salivary proteins or glycoproteins fill the space between the teeth and the aligners, they can easily adhere to the teeth, forming an acquired cellular film on which bacteria are highly susceptible to colonizing, which in turn leads to the development of enamel white staining lesions (WSLs), one of the major complications of orthodontic treatment. Inhibiting the activity of cariogenic bacteria while promoting the remineralization of demineralized enamel is the key to preventing and treating WSLs. Currently, the drug commonly used in clinical practice for the treatment of WSLs is silver diamine fluoride, which, although it has both antimicrobial and remineralizing effects, suffers from problems such as pulpal irritation and tooth discoloration. In this study, based on the principle of coordination chemistry, copper ions and plant polyphenol tannins were assembled on invisible orthodontic aligners to form a metal–phenol network coating (TA-Cu MPNs), and zwitterionic sulfonamethyldopamine was introduced for bionic mineralization to obtain the multifunctional coating TA-Cu MPNs@ZDS@CaP (TZC). The coating exhibits acid-responsive release of Ca^2+^ and PO_4_^3−^, and the decomposed CaP layer can be regenerated by a simple dipping method. The TZC coating strongly inhibits common cariogenic bacteria and their biofilms. In addition, the results of the *in vitro* mineralization experiment show that TZC-coated invisible orthodontic aligner treatment of demineralized enamel has significant remineralization effects. It is worth mentioning that the constructed coating has a durable antibacterial effect and can meet the service cycle of invisible orthodontic aligners. This study provides theoretical and experimental bases for the prevention or treatment of WSLs in invisible orthodontic treatment.

## 1 Introduction

With the improvement of living standards and the continuous pursuit of esthetic appearance, an increasing number of people are paying attention to dental health and appearance (Sischo and Broder, 2011). The number of individuals participating in orthodontic treatment has been growing annually. As one of the main methods of orthodontic treatment, nonbracket invisible orthodontics is becoming increasingly popular because of its relative esthetics and comfortable wear (Djeu et al., 2005; Shrivastava et al., 2023). However, invisible orthodontic aligners also present some issues that cannot be ignored. For instance, when wearing an invisible orthodontic aligner, salivary proteins or glycoproteins fill the space between the teeth and the aligner. They can readily adhere to the teeth, forming an unstructured acellular membrane called the acquired pellicle (Ayoub et al., 2020). Once the acquired pellicle is formed, bacteria can subsequently colonize it. To a certain extent, invisalign orthodontic aligners hinder the sterilizing effect of saliva, which further promotes the rapid division, reproduction, and growth of colonized bacteria (Lynge Pedersen and Belstrøm, 2019). This scenario increases the risk of developing white spot lesions (WSLs) on the dental enamel, with a prevalence exceeding 19% in the population (Xu et al., 2020). WSL affects the esthetic appearance of teeth and progresses into enamel caries (Marinelli et al., 2021). In orthodontic treatment, cariogenic bacteria in the oral cavity are the primary cause of WSLs. Therefore, effectively preventing and treating WSLs, inhibiting the activity of cariogenic bacteria, and promoting the remineralization of demineralized enamel is of utmost clinical significance.

The principle of preventing or treating WSLs is to halt the progression of demineralization, promote remineralization, and restore the structure and physiological function of teeth (Marinelli et al., 2021). Silver diamine fluoride (SDF) is a topical fluoride solution commonly used for treating caries in clinical practice. SDF has a dual effect of antibacterial activity and remineralization. After the use of SDF, less soluble or almost insoluble calcium fluoride, silver phosphate, and silver protein were formed and precipitated on the dentin surface to form an insoluble protective layer and reduce the loss of calcium and phosphorus from caries (Rosenblatt et al., 2009; Mei et al., 2018). However, SDF poses several safety concerns in clinical applications, such as the cytotoxicity of silver ions and fluoride. In addition, the use of SDF is associated with several adverse reactions, including pulp irritation, tooth discoloration, and irritation of oral soft tissues (Seifo et al., 2020; Xu et al., 2024). Therefore, the development of new and effective methods for the prevention and treatment of WSLs is crucial.

Surface modification is an effective method for introducing desired functionality into the surface of biomedical devices, providing additional functionality without compromising their bulk properties (Han et al., 2021). Metal–phenolic networks (MPNs) are a novel surface modification strategy that can be prepared using a facile metal–phenolic assembly method and have been developed in recent years (Ejima et al., 2013; Guo et al., 2014; Zhong et al., 2018; Yun et al., 2019). MPNs have received much attention in the field of antimicrobial infections because of their excellent biocompatibility and superior antimicrobial properties brought on by polyphenols and metal ions (Yao et al., 2024). In addition, MPNs can be deposited on almost any substrate in an aqueous environment because of the multifunctional chemistry of the catechol and gallol groups. The synthesis process is quite robust with respect to concentration, phenolic ligand selection, and metal ion selection. To date, libraries of functional coatings have been generated for a wide variety of applications (Fan et al., 2022; Li et al., 2022). However, the application of MPNs in the construction of multifunctional orthodontic aligner coatings has rarely been reported.

In the realm of remineralization, a method known as biomimetic mineralization, which emulates the natural mineralization process, has provided a novel approach to the restoration of demineralized hard tissues in teeth (He et al., 2022). Materials with remineralization-promoting properties can generally be divided into two categories. One category involves modifying the enamel or dentin surface with polymer films, inducing hydroxyapatite (HAp) mineralization directly on the carious lesions. Examples include polyamidoamine (Chen et al., 2014) and polyethylene glycol–coupled lysozyme (Li et al., 2019). This method relies on saliva providing supersaturated Ca^2+^ and PO_4_^3−^. However, the efficiency of this method in guiding mineralization is limited because of the direct correlation between the amounts of Ca^2+^/PO_4_^3−^ and the nucleation of HAp, especially in patients with xerostomia. Another method involves the use of polymer additives such as casein phosphopeptide (Cochrane et al., 2008; Reema et al., 2014), polyaspartic acid (Thula et al., 2011; Babaie et al., 2021), and polyacrylic acid (Ali et al., 2022) to stabilize the precursor phase and facilitate the permeation of precursor ions. During the mineralization process, these additives mediate the transformation of the mineral phase from ACP to the crystalline phase HAp. In recent years, zwitterionic materials have gained widespread attention because of their unique structural characteristics and excellent biocompatibility (Venault and Chang, 2019). These materials have both positively and negatively charged components, resulting in an overall neutral charge. Because of ion hydration, zwitterionic materials can firmly hold water molecules on the surface, forming an almost crystalline water layer, thus preventing nonspecific adsorption. He et al., 2022 used a zwitterionic polymer, carboxybetaine acrylamide (PCBAA), known for its antibacterial and antifouling properties, to stabilize ACP (He et al., 2022). They discovered that the neighboring amino groups (–R_3_N^+^) and carboxyl groups (–COO^−^) in PCBAA could serve as precipitation sites for Ca^2+^ and PO_4_^3−^, thereby reducing the oversaturation of mineral ions in the solution and increasing the likelihood of amorphous mineral crystallization.

**SCHEME 1:**
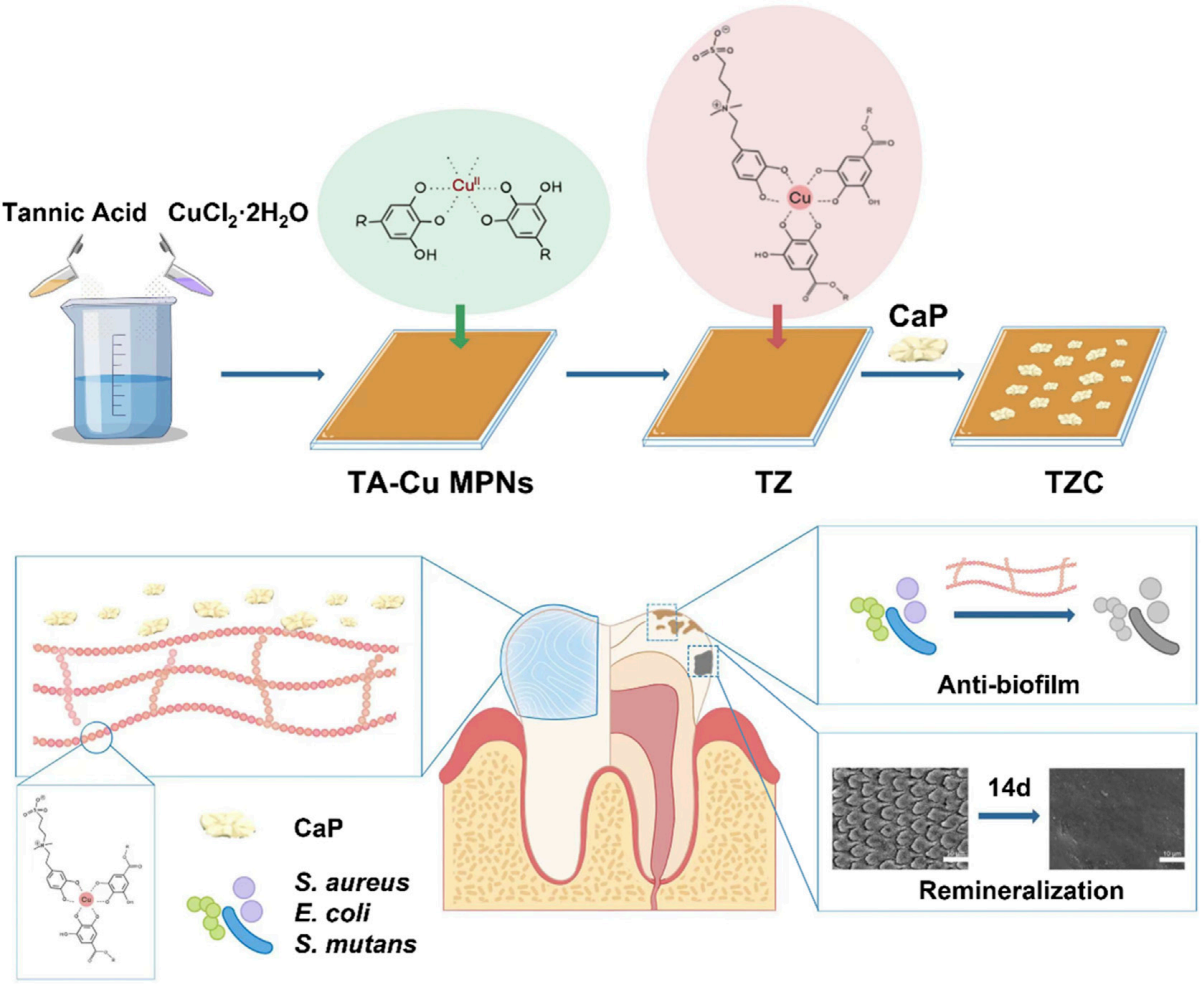
Schematic illustration of the preparation of the TZC coating and its antimicrobial properties and remineralization function.

Based on the above discussion, a novel pH-responsive invisible orthodontic aligner coating for preventing and treating WSLs was proposed. The strategy aims to achieve a multifunctional and comprehensive anti-WSL effect while being convenient to use. As shown in Scheme 1, tannic acid (TA) was used as an MPN to form TA-Cu MPNs on orthodontic aligner diaphragms. Then zwitterionic dopamine sulfonate (ZDS), with outstanding antibacterial properties, was introduced on the TA-Cu MPNs. Finally, calcium phosphate (CaP) was loaded onto the surface of TA-Cu MPNs@ZDS (TZ) by biomimetic mineralization to form the composite nanocoating TA-Cu MPNs@ZDS@CaP (TZC). The following hypotheses were tested. 1) Under acidic conditions generated by biofilm infection, the renewable CaP coating of TZC degraded and released Ca^2+^ and PO_4_^3−^ and then exposed the naturally active antimicrobial ZDS, which has a good destructive effect on cariogenic biofilm. 2) The released Ca^2+^ and PO_4_^3−^ can promote the self-repair of teeth and effectively stabilize and restore the hard tissue of teeth. The invisible orthodontic aligner coating with the dual action of antibacterial and remineralization has great clinical application potential to reduce the incidence of orthodontic white spots and prevent dental caries.

## 2 Materials and methods

### 2.1 Materials

Calcium chloride dihydrate (CaCl_2_·2H_2_O) and disodium hydrogen phosphate (Na_2_HPO_4_) were purchased from Aladdin. TA (ACS reagent), dopamine hydrochloride, and Trizma hydrochloride (Tris) were purchased from Sigma-Aldrich. Phosphate buffered saline (PBS), 1,3-propanesultone, and the LIVE/DEAD BacLight Bacterial Viability Kit were purchased from Solarbio. All other chemicals and solvents were analytical reagents and could be used without further purification.

### 2.2 Synthesis of ZDS

The syntheses of ZDS follow those developed in previous work with some modification (Wei et al., 2021). Specifically, dopamine hydrochloride (2.2752 g, 6 mmol) was dissolved in 220-mL ethanol. The flask was evacuated and filled with N_2_, and then ammonium hydroxide (28%, 832 µL) and 1,3-propanesulfonate (855 mg, 7 mmol) were slowly added. The solution was heated to 55°C and stirred for 18 h. Subsequently, the mixture was filtered, and the solid product was collected and purified by washing with ethanol three times. Dopamine sulfonate was dried under reduced pressure and characterized using nuclear magnetic resonance (NMR, Bruker DRX 400, Germany).

Dopamine sulfonate (0.3286 g, 1 mmol) was dissolved in 150 mL of dimethylformamide (DMF) in a 500-mL round-bottomed flask. Anhydrous sodium carbonate (0.2544 g, 2.4 mmol) was added to the DMF solution. Initially, sodium carbonate may not have completely dissolved. Then, iodomethane (2.2 mL, 35 mmol) was added. The solution was stirred at 50°C for 5–10 h. At this point, sodium carbonate was completely dissolved, and the reaction mixture turned yellow upon completion of methylation. DMF was removed using a rotary evaporator at 40°C, and an oily mixture was obtained. Subsequently, 50 mL of DMF/ethyl acetate (1:10 v/v) was added, and the yellowish crude product was precipitated. After filtration, 50 mL of DMF/acetone (1:10 v/v) was added to the crude product and refluxed at 55°C for 2 h. The solution mixture was filtered once again, and the precipitate was collected. These refluxing and filtration processes were repeated twice to obtain a white solid, which was characterized by NMR, indicating that it was pure ZDS.

### 2.3 Binding capacity of ZDS and Cu^II^

We used UV–vis spectroscopy to examine the binding capacity of ZDS and Cu^II^. All stock solutions were freshly prepared before measurements and degassed by continuous purging with nitrogen for 3 h in a closed atmosphere. ZDS was dissolved in 10 mM Tris buffer at a stock concentration of 0.1% (w/v). A 20 mM solution of metal Cu^II^ was prepared in oxygen-free distilled water. Cu^II^ solutions at concentrations ranging from 0.01 to 0.08 mM in Tris buffer were prepared before measurements. The ZDS stock solution was added to the copper solutions for a final concentration of 0.001% (w/v) throughout the measurements.

### 2.4 Fabrication of TA-Cu MPNs@ZDS@CaP (TZC) coating

First, 12.5 mg of TA, 20 mg of CuCl_2_·2H_2_O, and 50 mL of deionized water were mixed in a beaker. Subsequently, the pH of the mixture was adjusted to eight using a 1 M NaOH aqueous solution. The aligner diaphragms (2.5 × 2.5 cm) were then immersed in the aforementioned solution for 12 h. After rinsing with deionized water and drying under nitrogen for 24 h, sheets with the TA-Cu MPN coating were obtained. Second, these sheets were treated with CuCl_2_·2H_2_O (0.74 mg/mL) and ZDS solution (2 mg/mL) in Tris buffer solution for 10 min and 9 h at room temperature, respectively. Sheets with TA-Cu MPNs@ZDS (TZ) coating were obtained after washing and nitrogen-drying. Finally, the TZC coating was fabricated using the alternating dipping method (Zhou et al., 2022). The sheets were immersed in CaCl_2_ solution (500 mM), water, and Na_2_HPO_4_ solution for 30 s. This alternative impregnation process was repeated for 8–10 cycles to induce CaP coating on the TZ to obtain TZC.

### 2.5 Characterization

The successful synthesis of dopamine sulfonate and ZDS was confirmed through NMR. The surface morphology and element mapping of the coatings were characterized by scanning electron microscopy (SEM, VEGA3, TESCAN, Czech Republic) equipped with energy dispersive spectrometry (EDS). The thickness of the coating was measured using an ellipsometer (Gaertner LSE Stokes). Surface roughness was detected by atomic force microscopy (AFM, SPI 3800, NSK Inc., Japan). The chemical composition of the coating was analyzed by X-ray photoelectron spectroscopy (XPS, K-Alpha, Thermo Electron). Static water contact angles were evaluated by a contact angle goniometer (Phoenix mini, Surface Electro Optics, Seoul). The microsyringe produces droplets of 5 μL, and each sample was measured in triplicate.

### 2.6 *In vitro* pH responsiveness

The orthotic diaphragm coated with TZC were placed in an aqueous solution with pH levels of 5.5 and 7.4. After 5, 15, and 30 min, the aligner diaphragms were removed and dried under a nitrogen atmosphere for 12 h before SEM observation. In addition, the orthotic diaphragm coated with TZC were immersed respectively in aqueous solution at pH 5.5, 7.4, and 8.5 for 30 min. The release of Ca^2+^ from TZC was measured by inductively coupled plasma emission spectrometer.

### 2.7 Regeneration properties of TZC

The prepared patch of the appliance containing TZC was immersed in 500 mL of aqueous solution (pH 5.5) for 30 min. After drying in a nitrogen environment, the patch of the appliance was impregnated alternately in CaCl_2_ solution, deionized water, and Na_2_HPO_4_ solution for 30 s, and the alternating impregnation process was repeated for 8–10 cycles. The surface morphology was observed after drying in a nitrogen environment.

### 2.8 Antibacterial and antibiofilm performance

#### 2.8.1 Bacterial cultivation

In this experiment, *Streptococcus mutans* (*S. mutans*, mainly responsible for dental caries, strain BNCC 336931) was cultured at 37°C in brain heart infusion medium. *Staphylococcus aureus* (*S. aureus*, strain ATCC 6538) and *E. coli* (*Escherichia coli*, strain ATCC 25922) were cultured at 37°C in Luria–Bertani (LB) broth.

#### 2.8.2 Antiplanktonic bacterial viability of TZC

Test inoculum at a concentration of approximately 6 × 10^5^ cells/mL was first prepared, and the aligner diaphragm was UV-sterilized before and after coating and placed in sterile culture dishes. Then, 0.1 mL of test inoculum was distributed on the surface and covered with a piece of film (20 × 20 mm, made of polyethylene, polypropylene, or polyethylene terephthalate). The air between the surface and the film was gently squeezed out, and the test inoculum was spread to the edges. It was then incubated at 37°C and >90% relative humidity for 24 h. Finally, the surviving bacteria on the samples and membranes were collected into new sterile Petri dishes by washing with sterile saline. A 10-fold serial dilution of the eluted bacteria was prepared and spread on agar to count the colonies. The antimicrobial rate was determined using the following equation:

$$\text{AR}=\left(N_{0}\;‐\;N\right){/N}_{0}\times 100\%$$

where AR represents the antimicrobial rate and N_0_ and N are the number of colonies on the control and coated samples, respectively.

In addition, to explore the antibacterial mechanism of the coating, we immersed the aligner diaphragm loaded with the coating and the original diaphragm in the bacterial suspension (6 × 10^5^ cells/mL) for 24 h, and counted the live bacteria in each suspension by the coated plate method.

#### 2.8.3 Antibiofilm performance

The aligner diaphragms with the TZC coating were placed into the bottom of the laser confocal dish, and 2 mL of bacteria suspension (1 × 10^6^ CFU/mL) was added. After incubation in an anaerobic environment at 37°C for 48 h, the coculture medium was removed. The formed biofilms were then rinsed gently with PBS three times and stained using the LIVE/DEAD BacLight Bacterial Viability Kit in the dark for 30 min. Fluorescence images were obtained by confocal laser scanning microscopy (CLSM, SP8, Leica Microsystems), and the biofilm thickness was measured using ImageJ. NucGreen is a nucleic acid dye that causes bacteria with intact cell membranes to appear green, whereas dead bacteria with damaged cell membranes can only be stained with the red nucleic acid dye EthD-III. The aligner diaphragm without the coating was used as a blank control group. All determinations were conducted in triplicate experiments.

Biofilm biomass was then detected by the crystal violet (CV) staining. The bacterial suspension (1 × 10^6^ CFU/mL) was inoculated into a 6-well plate with the aligner diaphragms with the TZC coating as the bottom and incubated at 37°C for 48 h. The formed biofilms were then gently rinsed three times with sterile PBS and stained with 0.1% (w/v) CV staining solution for 30 min. The biofilm staining images were captured and recorded. After removing the CV dye and gently washing with sterile PBS, the biofilms were digested with 95% alcohol for 1 h. The biomass of the biofilm was quantified by recording the absorbance at 600 nm.

#### 2.8.4 Antibacterial durability of TZC

The aligner diaphragms with the TZC coating were placed into the bottom of a six-well plate, and 2 mL of bacterial suspension (10^6^ CFU/mL) was added to the coculture for 48 h to form biofilms. After cleaning the aligner diaphragms with PBS solution to remove unbound bacteria, the CV-stained biofilms were digested with 95% alcohol for 1 h. The biofilms were thoroughly shaken and diluted, and absorbance was measured at 600 nm to characterize the biofilm biomass. Subsequently, residual extracellular matrix and bacteria were removed from the surface by ultrasonically washing the aligner diaphragms in PBS for 10 min. We repeated these steps using the same aligner diaphragms containing the coating to verify its antimicrobial durability.

### 2.9 Cytotoxicity assay

The *in vitro* biocompatibility of the coatings was evaluated using the cell counting kit-8 (CCK-8) method. Primary human gingival fibroblasts (hGFs) were obtained from the gingival tissues of adolescents after alveolar surgery at Qingdao Dental Hospital. The following assay was performed using hGFs of the fourth to sixth generations: aligner diaphragms containing different coatings (cut into disks with a diameter of 6 mm) were placed in sterilized 96-well plates, and the cells were inoculated on each disk and incubated at 37°C for 48 h. After removing the supernatant and incubating with 100 µL of DMEM containing 10% CCK-8 (Dojindo, Japan) per well, the mixture was incubated at 37°C for 2 h. The absorbance of each well was recorded at 450 nm. The percentage of cell viability was determined using the following equation:

$$\text{Cell viability }\left(\%\right)=\left[A450\;\left(\text{experimental}\right)\;‐\;A450\;\left(\text{medium}\right)\right]/\left[A450\;\left(\text{control}\right)\;‐\;A450\;\left(\text{medium}\right)\right]$$

### 2.10 Hemolysis assay

Human whole blood was obtained from the Clinical Laboratory of Qingdao Stomatological Hospital and diluted to the final concentration (20%, v/v) with normal saline. The appliance patch (6-mm diameter disc), both uncoated and loaded with different coatings, was placed at the bottom of a 1.5-mL test tube and 50 μL of diluted blood as described above and 1 mL of normal saline was added. Triton X-100 (0.1%) was used as a positive control, and PBS was used as a negative control. Put the above experimental group and control group at 37°C environment to foster for 1 h. At the end of incubation, the solution was centrifuged at 1000 × *g* for 5 min and the hemolytic activity was determined at 540 nm with a microplate reader (BioTek ELX800, BioTek Instruments, United States).

### 2.11 Preparation of remineralization samples

Human third molars were obtained from 18- to 30-year-old patients who underwent prophylactic extractions at Qingdao Stomatological Hospital. The inclusion criteria were mature teeth without cavities, cracks, or other defects. The crown was separated from the teeth at the cement–enamel junction, and enamel slices were cut using a low-speed, water-cooled diamond saw to obtain 4 × 3 × 1 mm^3^ samples. Enamel slice samples were etched with 37% phosphoric acid for 30 s to produce a demineralized model, rinsed well with deionized water, and sonicated for 5 min (He et al., 2022).

### 2.12 *In vitro* enamel remineralization assay

To assess their potential role in enamel remineralization, artificially prepared enamel demineralization models were processed using aligner diaphragms with TZC coatings. An early demineralized enamel damage model was created by acid etching the surface of human tooth enamel sections with 37% H_3_PO_4_ for 30 s. The enamel sections obtained were placed on aligner diaphragms containing coatings (n = 12), which were placed together in 10 mL of artificial saliva, replaced daily with fresh artificial saliva, and incubated at 37°C for 2 weeks. After rinsing several times with deionized water and drying, the surface morphology was observed by SEM, the elemental distribution was analyzed using the X-ray energy spectroscopy elemental image analysis technique, and the crystal orientation of the enamel surface was analyzed by X-ray diffraction (XRD, Rigaku Ultima IV, Japan) to identify crystal structures. The samples were examined between 10° and 60° (2*θ*) at a scanning rate of 5° (2*θ*) per minute equipped with Cu Kα radiation (λ = 0.15418). Fourier transform infrared (FTIR) spectroscopy analyses of materials for each group in the range of 400–4000 cm−1 were performed with an attenuated total reflectance-infrared (ATR-IR) system by a Nicolet iN10 FTIR spectrometer (Thermo Fisher Scientific, Waltham, MA, United States).

### 2.13 Statistical analysis

All data are presented as the average value ±standard deviation of the independent experiment. A single-element variance analysis was adopted to conduct multiple comparisons. All related statistical analyses were from at least three parallel experiments, without any other special explanation: *p < 0.05, **p < 0.01, and ***p < 0.001.

## 3 Results and discussion

### 3.1 Binding capacity of ZDS and Cu^II^

**FIGURE 1:**
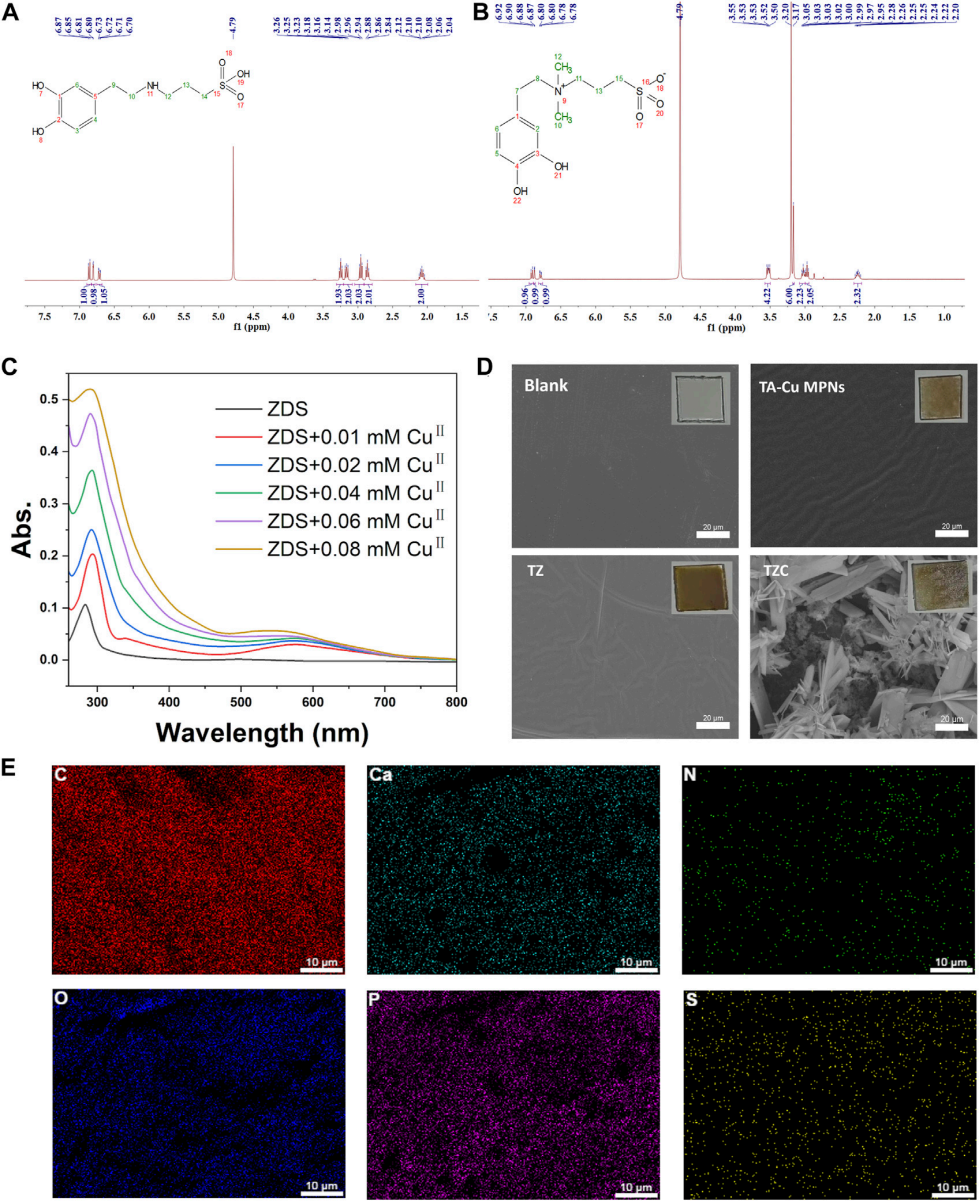
**(A)** ^1^H NMR of DS and **(B)** ZDS (the peak at *δ* = 4.80 was from D_2_O). **(C)** UV–vis spectra for coordinating ZDS with CuII. CuII solutions at concentrations ranging from 0.01 to 0.08 mM in Tris buffer were mixed with ZDS. **(D)** Surface morphologies of the TA-Cu, TZ, and TZC coatings were determined by scanning electron microscopy (SEM). Insets are macrophotos of different coatings. **(E)** Elements distribution of TZC.

ZDS was synthesized according to a previous study (Wei et al., 2012), and its chemical structure was confirmed by ^1^H NMR spectroscopy (Figures 1A, B). The interaction between ZDS and Cu^II^ was investigated using UV–vis spectroscopy. As shown in Figure 1C, the UV–vis spectrum of ZDS has a maximum absorbance peak at λ = 280 nm. After the addition of Cu^II^, a broad band was detected in the range of λ = 500–700 nm, indicating the formation of a phenol–copper complex. In addition, a bathochromic shift of the characteristic absorbance band with increasing copper concentration was observed in the region of 220–350 nm.

### 3.2 Characterization of TZC coatings

**FIGURE 2:**
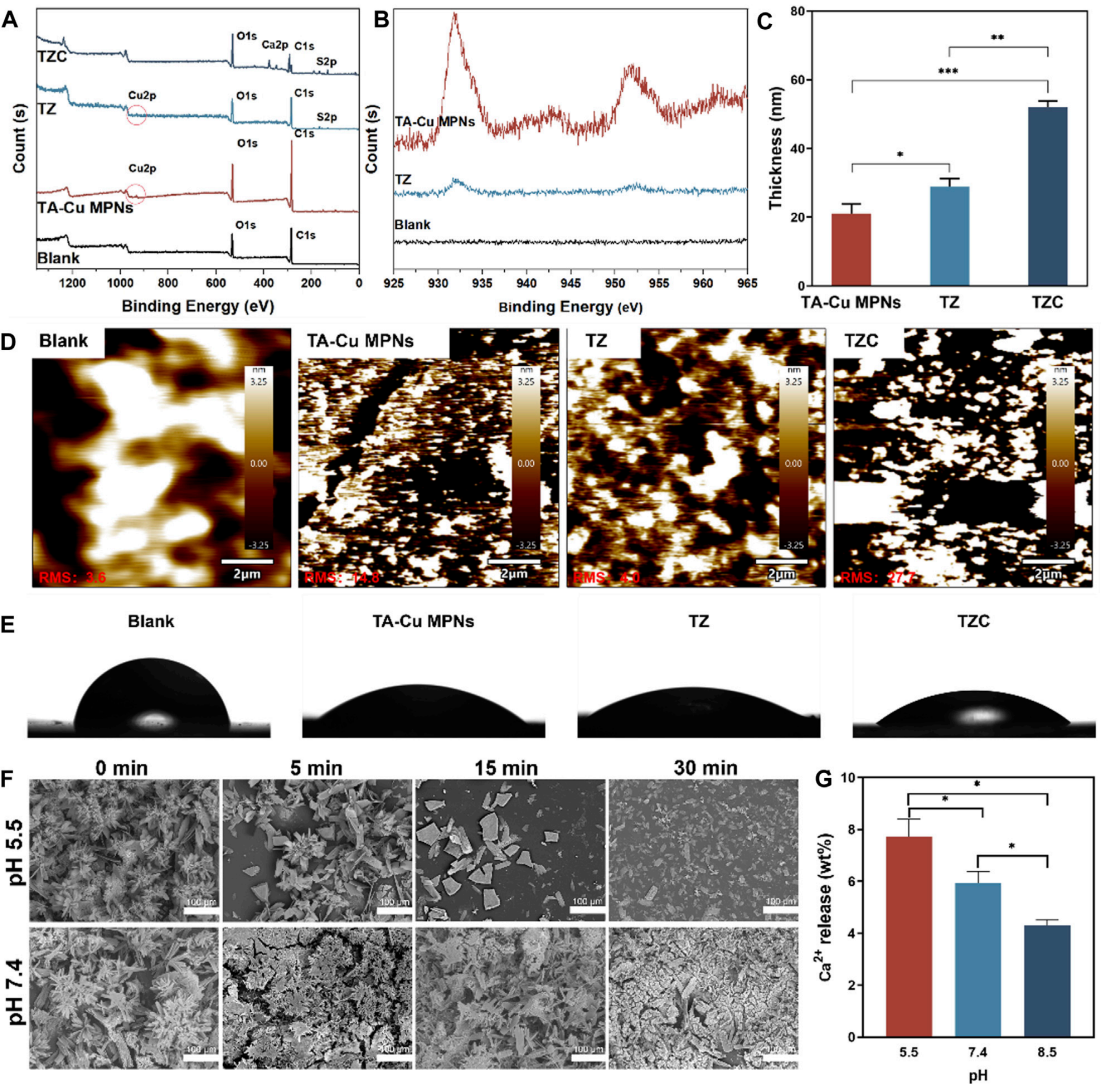
**(A)** XPS spectra of the TA-Cu MPNs, TZ, and TZC coatings. **(B)** Copper XPS fine spectra of the TA-Cu MPNs and TZ coatings. **(C)** Thickness evolution of the TA-Cu MPNs, TZ, and TZC coatings as measured by spectral ellipsometry. **(D)** Roughness of the TA-Cu MPNs, TZ, and TZC coatings. The root mean square (RMS) roughness was determined by AFM from images with a scanning area of 20 × 20 μm^2^. **(E)** Water contact angle measurements for the TA-Cu MPNs, TZ, and TZC coatings. **(F)** pH responsiveness of the TZC coatings: the morphology of the TZC coatings at different times (pH = 5.5 and 7.4). **(G)** Ca^2+^ content released within 30 min of TZC coating at different pH values.

The synthesis process for the TZC coating is shown in Scheme 1. First, TA-Cu^II^ MPNs were deposited on aligner diaphragms after immersion for 12 h in an alkaline solution containing tannic acid (TA) and CuCl_2_·2H_2_O. The aligner diaphragms showed a typical brown color of polyphenol, confirming the effectiveness of the Cu^II^–phenolic surface chemistry strategy. Then, ZDS was bound through coordination with Cu^II^ to form the TZ coating. Finally, a CaP coating was constructed on the surface of TZ by biomimetic mineralization to form TZC (Li et al., 2023). Next, the morphologies of the fabricated coating were observed. As shown in Figure 1D, the TA-Cu MPN, TZ, and TZC coatings were brown overall; therefore, we recommend wearing clear aligners loaded with coatings at night during clinical use. In addition, because of the lack of oral activity at night, salivary protein and glycoprotein are more likely to fill the gap between the appliance and the teeth and form an acquired biofilm. Wearing coated aligners at night can effectively prevent WSLs. The TA-Cu MPN and TZ coatings exhibited a smooth surface with some wrinkles compared with the blank aligner diaphragm. The surface of TZC was clustered with patterned lamellar structures. The energy spectrum shows the distribution of the six elements (Ca, P, C, N, S, and O; Figure 1E). In addition, the element composition and chemical state of the TZC coating on the aligner diaphragm were analyzed by XPS (Figures 2A, B). One distinct component at 925–965 eV, corresponding to Cu, was found in the TA-Cu MPN sample. This suggests that the signals were associated with a large number of unpaired copper ions on the MPN coatings (Ko and Huang, 2020). The TZ samples did not show an evident peak, which may be due to the zwitterionic catecholic DS being attached to the surface by chelating with Cu ions in the MPN coatings. In addition, the final TZC coating thickness was approximately 50 nm (Figure 2C), which was significantly different from those of the TA-Cu MPNs and TZ coatings. The root mean square (RMS) results of AFM showed that the surface roughness of the coatings was reduced with the introduction of ZDS into the coatings relative to TA-Cu MPNs, which may be attributed to the ionic hydration of the amphiphilic material. The roughness of the TZC coating increased because of the mineralization of CaP (Figure 2D). Results of static water contact angle (WAC) measurements were shown in Figure 2E. Aligner diaphragm without the loaded coating had a WAC of 83.05 ± 2.0°. After loading coatings, the WAC of TA-Cu MPNs, TZ, and TZC were 36.41 ± 1.69°, 29.00 ± 2.4°, and 38.28 ± 0.6°, respectively. This is a result of the hydrophilic groups on the surface of the coating and the high surface density of attached ZDS moieties. All of the above proved the successful synthesis of TA-Cu MPNs, TZ, and TZC.

**FIGURE 3:**
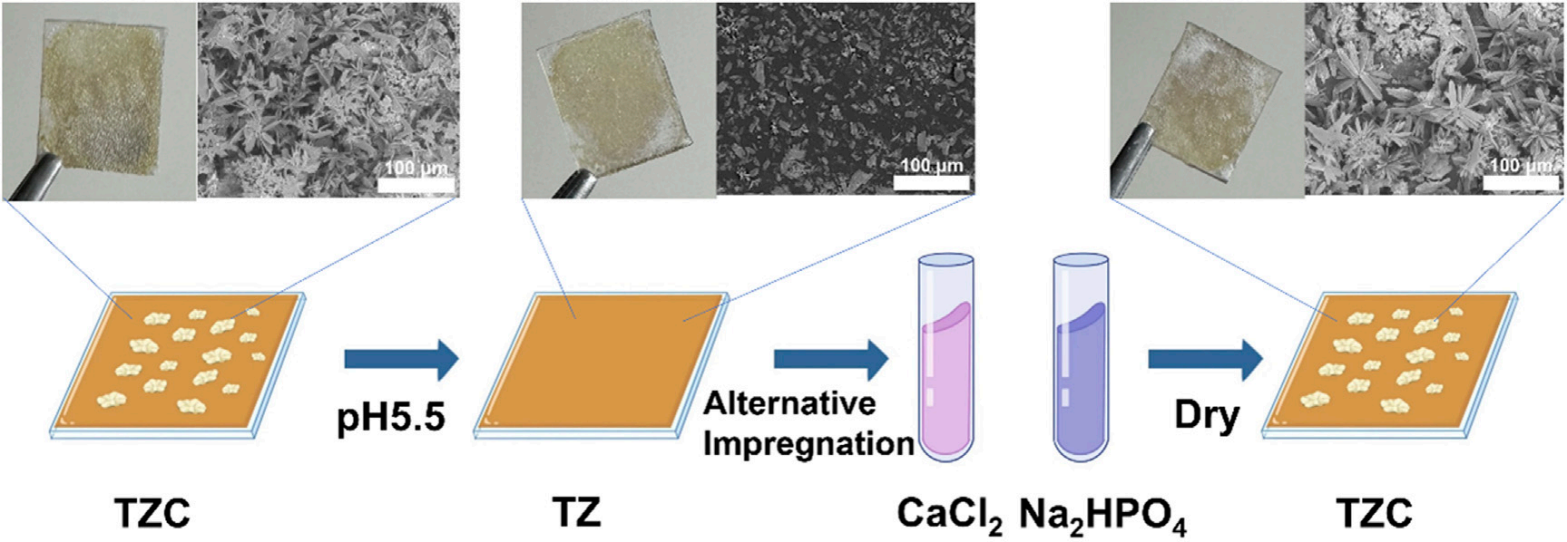
Schematic illustration and photographs of the TZC coatings before and after regeneration.

A low pH would demineralize the hard tissue structure of teeth (Yan et al., 2022; Dai et al., 2023). Therefore, the functional release of the coating can be triggered by pH signals that respond to this feature. To test the pH response of TZC coatings, it was placed in an aqueous solution with pH = 5.5 and 7.4 at different times, and the morphology of the coating was observed by SEM. The results showed that the CaP layer gradually decomposed within 30 min at pH = 5.5. At pH = 7.4, the CaP layer was relatively stable over time (Figure 2F). Correspondingly, at pH = 5.5, due to the decomposition of the CaP layer in the TZC coating, the TZC coating releases a large amount of Ca^2+^. For comparison, at pH = 7.4 and 8.5, TZC coatings also released Ca2+, but at a relatively low level (Figure 2G). In this process, we observed that with the dissolution of the CaP layer on the TZC coating surface, the coating color becomes closer to that of the TZ coating. Notably, the decomposed CaP coating was regenerated and recovered using a simple impregnation method. The schematics and photographs are shown in Figure 3. This property facilitates the long-lasting function of invisible aligners over their life cycle (∼10 days/pair).

### 3.3 Antimicrobial properties

**FIGURE 4:**
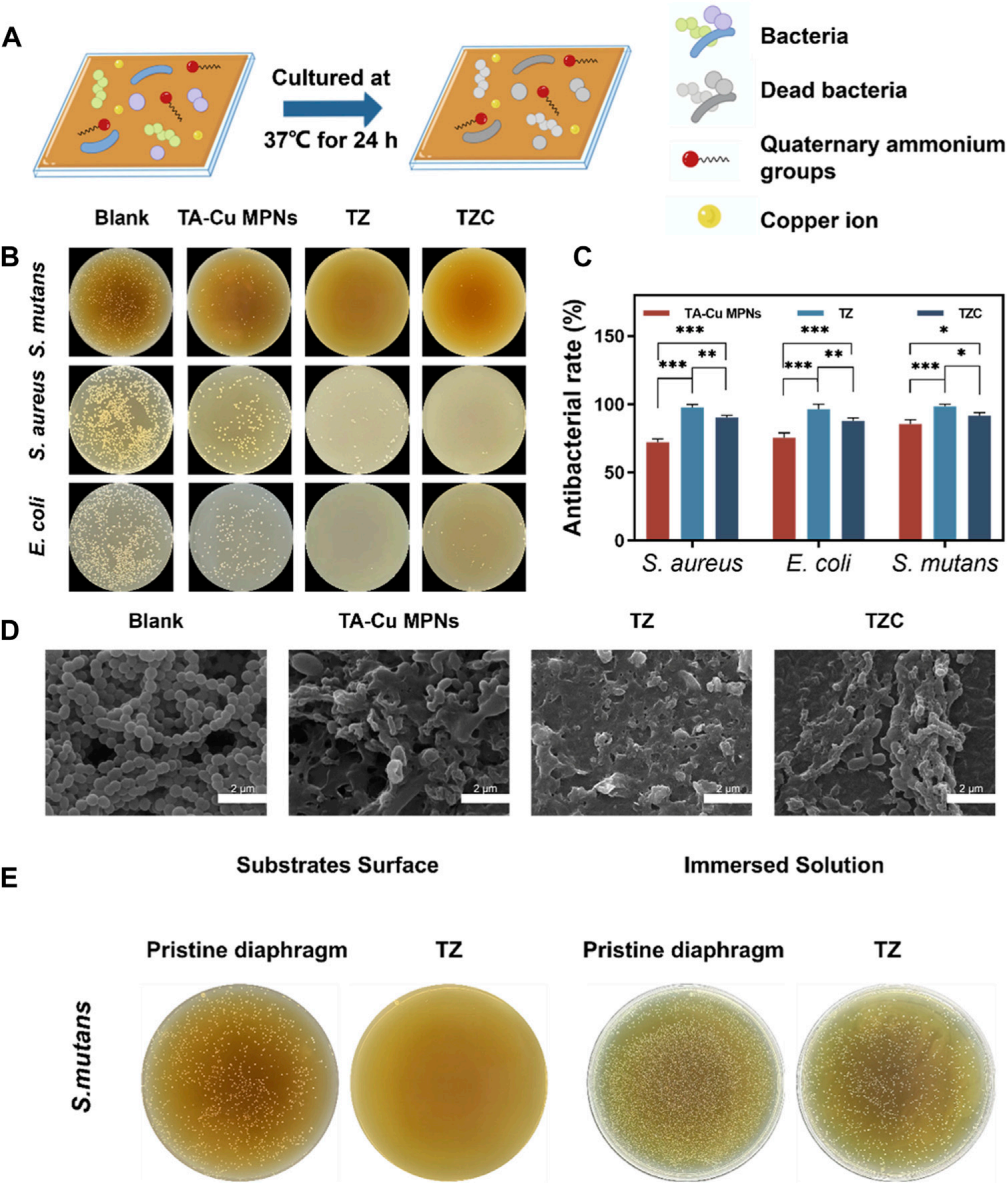
**(A)** Schematic illustration of antibacterial experiments on the TA-Cu MPN, TZ, and TZC coatings. **(B)** Antimicrobial efficiency of the TA-Cu MPNs, TZ, and TZC coatings. **(C)** Bactericidal rate of the TA-Cu MPNs, TZ, and TZC coatings. **(D)** SEM images of *S. mutans* biofilms on the TA-Cu MPNs, TZ, and TZC coatings. **(E)** Antibacterial properties of TZ coatings against attached and suspended bacteria.

When oral hygiene is not maintained well, the cariogenic bacterium metabolizes carbohydrates into organic acids, leading to enamel demineralization. Therefore, effective inhibition and destruction of the growth and reproduction of cariogenic bacteria is a focus of the prevention and treatment of WSLs. First, *S. mutans*, *E. coli* (Gram-negative bacteria), and *S. aureus* (Gram-positive bacteria) were selected for the antibacterial experiments (Figure 4A). As shown in Figures 4B, C, the TA-Cu MPNs could have a certain antimicrobial effect because of the presence of Cu^2+^ (Li et al., 2018). After the introduction of ZDS, the antimicrobial performance of TZ was further strengthened, and the bactericidal efficiency was close to 100% because of the synergistic antimicrobial action of positively charged quaternary ammonium groups and Cu^2+^. After forming the CaP layer, part of the positive charge is consumed, so the antibacterial effect of TZC is decreased.

Encouraged by the advantages of the constructed coatings, the number and morphology of adherent bacteria in each group of aligner diaphragms were investigated. We selected *S. mutans* as a model candidate. SEM was employed to characterize the morphological changes in bacteria. As shown in Figure 4D, large amounts of *S. mutans* colonies with obvious globular chain–like structures and intact cell membranes were observed on the surface of the uncoated aligner diaphragms. Under similar conditions, on the contrary, a few *S. mutans* remained on the surface of TZ. The bacteria appeared to have discernible structural distortion and collapse, or even complete lysis. In addition, compared with the results of previous experiments, after we immersed the corrective diaphragm loaded with TZ coating into the bacterial suspension, a large number of live bacteria were still present in the suspension (Figure 4E). Therefore, the main antibacterial mechanism of TZ coating is contact sterilization. The above experiments provide preliminary evidence of the desirable bacterial inhibitory activity of the coatings.

**FIGURE 5:**
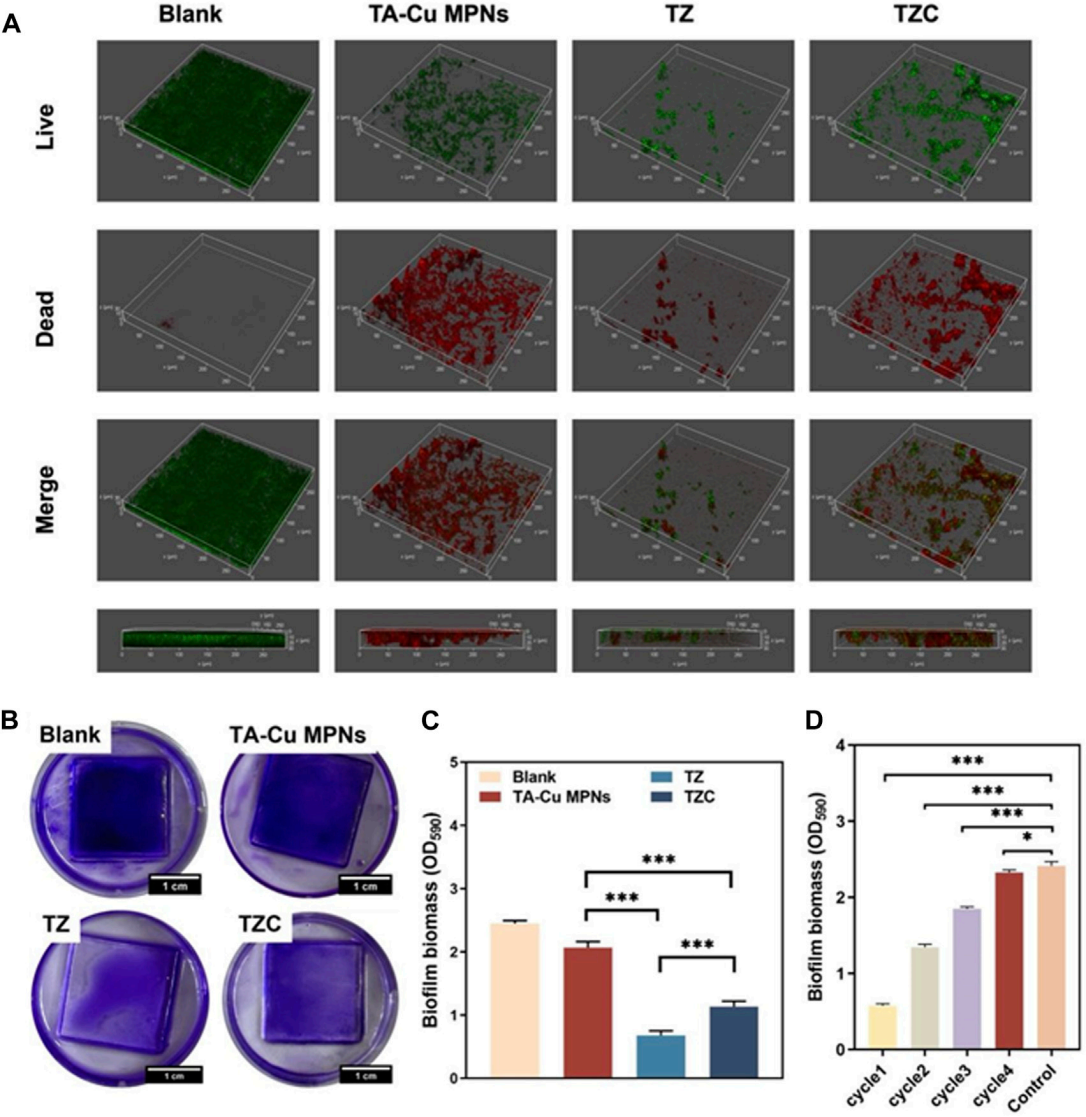
**(A)** Representative 3D live/dead staining images of *S. mutans* biofilms. **(B)** Crystal violet staining images of biofilm on the TA-Cu MPNs, TZ, and TZC coatings and **(C)** biofilm biomass (n = 3). **(D)** Histograms showing biofilm biomass on the TZC-coated aligner diaphragm with different cycle times.

The live/dead staining was performed to evaluate the antibiofilm effect (Figure 5A). After observation by CLSM, green and red fluorescence represents live and dead bacteria, respectively. In the uncoated aligner diaphragm group, the thick *S. mutans* was mainly green, and red fluorescence was not obvious. In the coating groups, many green spots appeared in the biofilm, suggesting that TA-Cu MPNs had an insufficient bactericidal effect on *S. mutans*. Almost no biofilm was formed on the TZ coating, and the residual biofilm showed strong red fluorescence, indicating that the zwitterionic quaternary ammonium cation effectively inhibited bacteriostatic adhesion to form the biofilm. In contrast, the inhibition ability of the TZC coating on the biofilm decreased slightly, indicating that the introduction of CaP weakened the antibiofilm performance of the TZC coating. However, the overall result was better than that of TA-Cu^II^ MPNs and the blank control group. Biofilm biomass after different treatments was quantified using CV staining. As shown in Figures 5B, C, all coatings provided antibiofilm effects, which were particularly noticeable in the TZ and TZC groups, with a significant reduction in biofilm biomass.

The antibacterial persistence of the TZC coating was tested. The biomass measurement results showed that the number of remaining biofilms increased gradually with an increase in the number of cycles, which may be related to partial coating loss (Figure 5D). However, in the fourth cycle, the TZC-coated aligner diaphragms did not inhibit biofilm formation. This extreme situation would not occur clinically because patients are generally instructed to rinse their mouth and aligners after their meals to prevent the adhesion and growth of bacteria, which is not conducive to biofilm formation.

### 3.4 *In vitro* enamel remineralization

**FIGURE 6:**
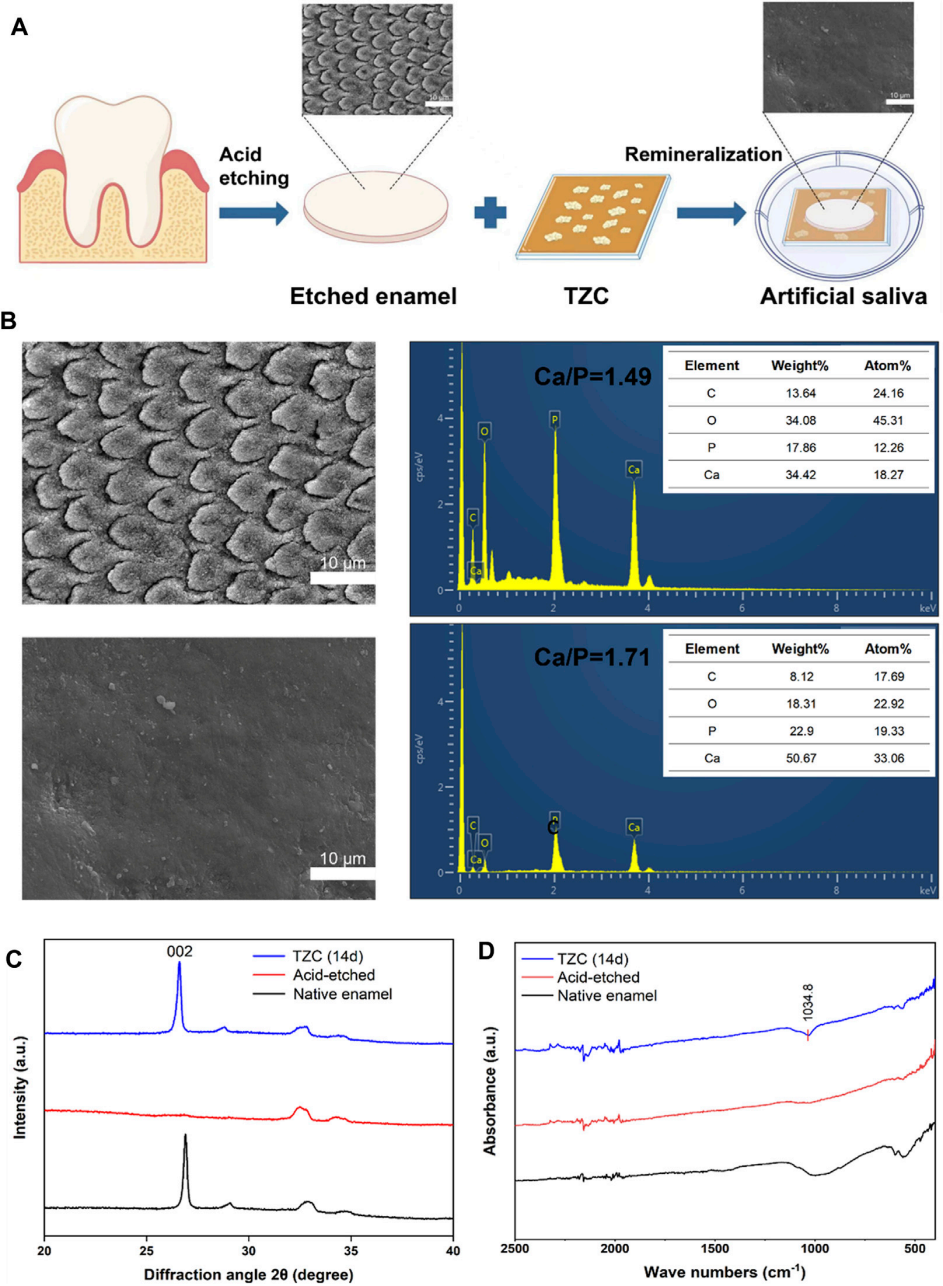
**(A)** Schematic illustration of the remineralization assay. **(B)** Electron micrographs of enamel sections after acid etching and Ca and P contents. **(C)** XRD analysis of native enamel, acid-etched, and TZC-incubated demineralized enamel after 14 days. **(D)** Infrared test result of native enamel, acid-etched, and TZC-incubated demineralized enamel after 14 days.

To assess their potential role in enamel remineralization, artificially prepared enamel demineralization models were processed in this experiment using aligner diaphragms containing coatings (Figure 6A). A model of early demineralized enamel damage was created by acid etching the enamel surface of slices of human teeth using 37% H_3_PO_4_ for 30 s. With regard to the effect of enamel remineralization, after 7 days of natural remineralization, the prismatic structure of the enamel surface in the blank group was exposed in a “fish scale” shape, and no significant remineralization layer was observed (Figure 6B). This is due to the dissolution of the organic and inorganic components of the enamel during demineralization. When HAp loses several mineral ions, the integrity of its lattice structure is destroyed, resulting in the formation of cavities. Although saliva has some ability to remineralize, its mineralizing effect is limited by the amount of calcium and phosphate ions in the saliva. In contrast, a model of enamel demineralization treated with an aligner diaphragm containing a TZC coating revealed a distinct mineral layer formed on the enamel surface, with the interstitial regions of the enamel columns nearly filled with newly formed crystals. These newly formed intermediate structures are most likely nanosized ACP particles that can easily penetrate the underglaze column region. The calcium and phosphorus ions provided by ACP thus increase its concentration in saliva, accelerating the transformation of the crystals to form HAp.

The type of newly formed crystals on the enamel was investigated by XRD. As shown in Figure 6C, the (002) diffraction peak at 2*θ* = 25.8 is characteristic of HAp and appears on healthy enamel as expected. After acid etching, the (002) characteristic peak was significantly weakened or disappeared. After 14 days of incubation in artificial saliva, the characteristic diffraction peak was clearly restored in the TZC-coated treated demineralized enamel, suggesting that crystalline HAp was newly produced on the samples. The Ca/P molar ratio in the natural enamel was close to the stoichiometric standard of 1.67 for HAp. EDS showed that the Ca/P molar ratio was reduced to 1.49 after acid etching because calcium and phosphorus ions experienced different proportional losses. After remineralization with the TZC coating, the Ca/P molar ratio increased to 1.71, which is close to the HAp molar ratio. The increased Ca/P molar ratio also confirms the XRD-based conclusion that the newly formed mineral on the enamel surface is HAp. As shown in Figure 6D, in the range 400–2500 cm^−1^, the characteristic band of PO_4_^3−^at 1090–1032 cm^−1^ (Reyes-Gasga et al., 2013) appears on healthy enamel as expected. After acid etching, the characteristic band of PO_4_^3−^ was significantly disappeared. After 14 days of incubation in artificial saliva, the characteristic band of PO_4_^3-^ can be found at the TZC-coated treated demineralized enamel. This result further proved that crystalline HAp was formed on the surface of the sample. In addition, we observed no significant color change in enamel sections during the experiment.

### 3.5 Cytotoxicity

**FIGURE 7:**
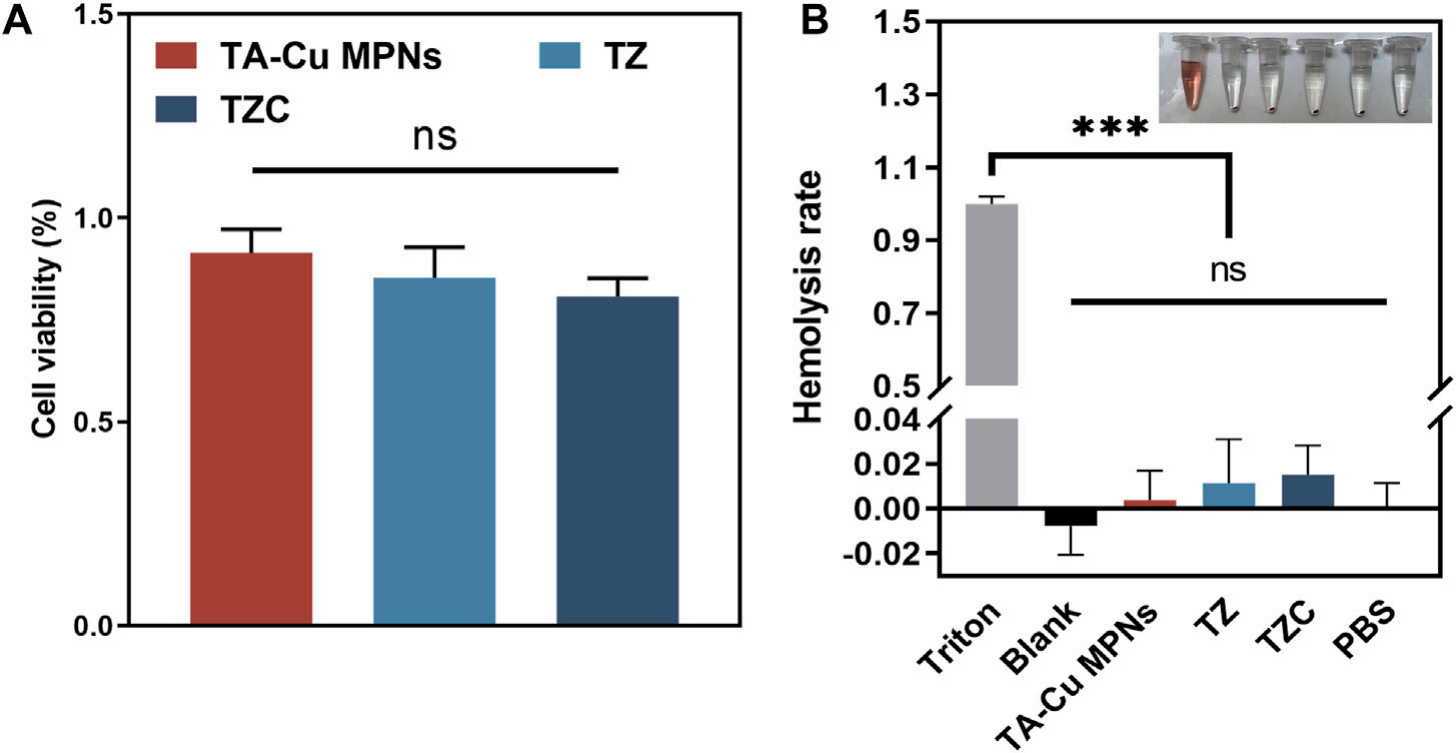
**(A)** Effect of the TA-Cu MPN, TZ, and TZC coatings on gingival fibroblast activity as determined by CCK-8. **(B)** Hemolysis assays of erythrocytes treated with different coatings.

Gingival fibroblasts were used to evaluate cytotoxicity. The CCK-8 method was used to study the effect of different coatings on cell viability. We used gingival fibroblasts cocultured with the aligner diaphragm as a control sample with 100% cell viability. Cell viability on the TA-Cu MPN surface was approximately 92%, indicating good biocompatibility. In contrast, positively charged quaternary ammonium groups increased the cytotoxicity of the TZ surface, and cell viability decreased to 85%. After the introduction of CaP, the cell viability on the TZC surface was approximately 81% (Figure 7A). Nevertheless, there was no statistical difference between the three groups. Overall, the coatings we have prepared were biocompatible with relatively low cytotoxicity.

### 3.6 Hemolysis assay

The results of blood tests (Figure 7B) indicated that red blood cells cultured in the experimental groups experienced little hemolysis, with a maximum hemolysis rate of <2%, complying with the ASTME2524-08 standard. Hence, TA-Cu MPNs, TZ and TZC showed good biocompatibility *in vitro*.

## 4 Conclusion

In summary, we have prepared a multifunctional coating with antibacterial and remineralization properties for invisible orthodontic aligners. ZDS was grafted onto TA-Cu^II^ MPNs. The introduction of ZDS not only provides excellent antibacterial properties but also provides binding sites for Ca^2+^ and PO_4_^3−^, which is conducive to biomimetic mineralization to form the CaP layer. In the acidic cariogenic microenvironment, the acid-responsive CaP layer can release a large amount of Ca^2+^ and PO_4_^3−^, promoting the remineralization of demineralized enamel. Notably, the dissolved CaP layer can be regenerated using a simple dipping method. The TZC coating has good durability against biofilm. TZC-coated invisible orthodontic aligners provide a promising prevention and therapeutic strategy to address clinical complications in orthodontic treatment.

## Data Availability

The original contributions presented in the study are included in the article/Supplementary Material, further inquiries can be directed to the corresponding authors.
